# Challenges of a Contact Investigation of Healthcare Personnel Exposed to Mycobacterium tuberculosis Contaminated Bone Allografts, 2023

**DOI:** 10.1017/ash.2025.370

**Published:** 2025-09-24

**Authors:** Yoran Sato, Janice Kim, Grace Kang, Yi-ning Cheng, Angela Miner, Romina Beltran, Therese Rymer, Debora Cartagena, Jeffrey Percak, Mark Beatty, Seema Shah, Ankita Kadakia, Juliet Stoltey, Erin Epson, Raymond Chinn

**Affiliations:** 1 California Department of Public Health; 2 County of San Diego, Health and Human Services Agency; 3 County of San Diego, HHSA, Public Health Services, TB Control & Refugee Health Branch; 4 County of San Diego, Health & Human Services; 5 County of San Diego TB Control and Refugee Health Branch; 6 County of San Diego Health & Human Services Agency, San Diego, CA; 7 County of San Diego - Public Health Preparedness and Response Branch; 8 County of San Diego; 9 County of San Diego HHSA; 10 California Department of Public Health, Healthcare-Associated Infections Program

## Abstract

**Background:** In response to a second multistate extrapulmonary tuberculosis (TB) outbreak in 2023, linked to contaminated viable bone allografts, public health authorities conducted contact investigations (CIs) to assess TB transmission among healthcare personnel (HCP) potentially exposed to contaminated grafts during surgeries or draining infected surgical sites. **Method:** A HCP-CI was initiated after Centers for Disease Control and Prevention (CDC) notification that three San Diego county hospitals had received contaminated allografts. Healthcare facilities identified and tested HCP potentially exposed and reported results to the health department. We reviewed the CI processes of each facility and outlined challenges encountered and solutions implemented. **Result:** HCP-CIs were conducted at five facilities: three hospitals where nine patients received contaminated product; a hospital where revision surgery was performed; and a skilled nursing facility (SNF) that provided postoperative care. We encountered several challenges during the CIs. First, 234 HCPs were potentially exposed based on a framework used in a prior (2021) TB bone allograft investigation. We advised a tiered approach with targeted follow-up of 72 HCP with high-risk exposures (for example, staff directly handling the allograft). Second, the SNF CI was complicated by administrative staff turnover, no SNF point-of-contact, and investigations involving different HCPs during three separate admissions. Substantial public health resources were required over 7 months, including a site visit to interview HCP with positive TB tests and obtain accurate CI information. Third, obtaining CI data was slow and inconsistent. Reasons included lack of a standardized data collection tool during the initial phase of the CI, fragmented information gathering across clinical departments within facilities, lack of responses from licensed practitioners who were not employees (e.g., physicians), variability in notification of exposed HCP for testing, and follow up of HCP that weren’t tested. HCP-CI results were not readily available when requested, leading to confusion, repeated requests, and duplication of efforts. We countered these challenges by leveraging established (or new) relationships with facility leadership and involving multidisciplinary staff to obtain results. Public health recommended facilities contact non-responsive practitioners with high-risk exposures by certified letter notifying them of the exposure and testing recommendations. **Conclusions:** Close collaboration, communication, and coordination between public health and each healthcare facility’s clinical services and leadership were critical in this HCP-CI. Utilizing a tiered approach streamlined the CI. This complex HCP-CI spanned multiple facilities and could have benefited from early identification of consistent points-of-contact at each facility and use of a standardized data collection tool.

